# Short fasting test as a reliable and effective tool to diagnose insulinoma

**DOI:** 10.1007/s12020-024-03759-7

**Published:** 2024-03-07

**Authors:** Nevena Mikovic, Rossella Mazzilli, Virginia Zamponi, Flaminia Russo, Camilla Mancini, Fedra Mori, Lucilla Bollanti, Francesco Conti, Cecilia Motta, Salvatore Monti, Giuseppe Pugliese, Antongiulio Faggiano

**Affiliations:** Unit of Endocrinology, Department of Clinical and Molecular Medicine, ENETS Excellence Center, Sant’Andrea Hospital, “Sapienza” University, Rome, Italy

**Keywords:** Insulinoma, Hypoglycaemia, Fasting test, Neuroendocrine neoplasm – NEN, Neuroendocrine tumours – NET

## Abstract

**Purpose:**

The diagnosis of insulinoma can be challenging, requiring documentation of hypoglycaemia associated with non-suppressed insulin and C-peptide, often achieved during a prolonged 72 h fast performed in inpatient setting. Our goal is to predict weather a shorter outpatient fasting test initiated overnight and prolonged up until 24 h could be a sensitive method for diagnosing insulinoma.

**Methods:**

We conducted a retrospective monocentric study on subjects admitted to our Unit of Endocrinology from 2019 to 2022 for clinical suspicion of insulinoma and underwent the short fasting test. A comparison between the short test group and the group of subjects who underwent the standard prolonged fasting test (from 2003 to 2018) has also been performed. The short fasting test was initiated by the patient overnight at home and proceeded the following day in outpatient setting (Day Hospital). As in the standard protocol, symptoms and capillary blood glucose (CBG) were strictly monitored. Venous blood was drawn for glycaemia, insulin and C-peptide at admission and at established intervals, in case of symptoms of hypoglycaemia or if CBG ≤ 45 mg/dl, when the fast would be suspended.

**Results:**

The final sample consisted of 37 patients, with mean age of 44.5 ± 12.6 years (17–74). Short and standard tests were performed in 15 and 22 subjects, respectively. Diagnostic values for insulinoma were observed in 12 patients: in 5/15 who underwent the short fasting test, in 6/22 who underwent the prolonged test and in 1 patient who was initially negative on the short test and subsequently showed diagnostic values during the prolonged test. The diagnosis of insulinoma was achieved in 11/12 cases within 24 h of the beginning of the fast (91.7%).

**Conclusions:**

A short fasting test could be a valid, sensitive and reliable first-line workup in diagnosing insulinoma.

## Introduction

Insulinomas are rare neuroendocrine tumours (NETs) with an estimated incidence of 1:250.000 individuals, with peak occurrence at 47 years, slightly more frequent in women and present in all ethnicities [1, 2]. These tumours derive from islet β-cells and are almost exclusively pancreatic but can rarely have ectopic localizations [3]. Clinical presentation consists in chronic hypoglycaemia with symptoms presenting mainly in the fasting period, which is caused by underlying endogenous hyperinsulinemia [2]. More rarely, they can also occur in the postprandial period [4]. Insulinomas almost always show benign behaviour with only about 6% being metastatic and are mostly single lesions, except when associated with Multiple Endocrine Neoplasia type 1 (MEN-1) syndrome, in which multiple lesions can often be found [1, 2, 4]. Most insulinomas can be treated and eradicated through surgical enucleation or pancreatectomy, while inoperable or metastatic diseases are candidate for systemic therapy or locoregional techniques [5–7]. Diagnosis requires strong clinical and biochemical evidence of endogenous hyperinsulinemia and negative screening for oral hypoglycaemic agents and insulin antibodies before attempting to perform tumour localization studies, since most tumours are <1 cm in diameter and therefore not always detected on traditional imaging methods such as CT or MRI [5]. Endoscopic pancreatic ultrasound with possibility of fine-needle aspiration of a detected nodule is an invasive method but has demonstrated a sensitivity of >90% [8, 9]. The Endocrine Society Clinical Practice Guidelines (ESCPG) define endogenous insulin-mediated hypoglycaemia as a plasma glucose concentration of <55 mg/dl combined with insulin ≥3 µU/ml (18 pmol/L), C-peptide ≥0.6 ng/mL (0.2 nmol/L) and Proinsulin ≥5.0 pmol/L, in association with signs and symptoms attributable to hypoglycaemia [10]. Symptoms are a result of neurogenic autonomic activation (such as tremor, sweating or palpitations) and neuroglycopenia (such as blurred vision, confusion or loss of consciousness) [11] and resolve when blood glucose levels arise, which defines the Whipple triad [12, 13]. When the spontaneous hypoglycaemic episode is not observed by clinicians, the supervised fasting test, which can be prolonged up until 72 h, induces hypoglycaemia in insulinoma patients and is the gold standard diagnostic protocol, with a sensitivity approaching 100% [13–15]. However, this test requires hospitalization and may be challenging to perform, potentially causing significant discomfort for the patient and requiring increased human and economic resources. In an attempt to overcome this obstacle, a 48-h maximum fast has been proposed by Hirshberg et al. [16] et al. with excellent sensitivity (95%), as well as other alternative shorter tests easier to perform in clinical practice [17–19] but further studies are needed to establish its standardized use.

The aim of this study is to determine whether a 24-h short fast performed in outpatient setting could be an adequate first-line evaluation in the diagnostic work-up of insulinoma.

## Methods

### Study design

In this retrospective monocentric study, we collected data from all patients who underwent a fasting test for suspected insulinoma at the Unit of Endocrinology of the Sant’Andrea University Hospital. The search was conducted through records of clinical files of our department ranging from 2003 to 2022. The protocol is compliant with the declaration of Helsinki and received approval from the local ethics committee (Rif. 7406. Prot. 0871/2023).

### Patients and procedures

All patients admitted to the Unit of Endocrinology who underwent a fasting test (standard 72 h test or short fasting test) for hypoglycaemia were included. All subjects had a clinical suspicion for insulinoma (documented hypoglycaemia in absence of other known causes for the symptoms and no current insulin therapy or assumption of hypoglycaemic drugs). Until the year 2018 the patients had undergone the 72 h fasting test. From 2019, due to insufficient availability of hospitalization to perform the prolonged test, the inpatient protocol was discontinued and replaced with the shortfasting test as first-line evaluation; in case of negative short test with remaining high clinical suspicion for insulinoma, it would be followed by the prolonged test. The 72 h fast was performed in line with ESPCG recommendations [10], with minimal variations: patients were admitted to the hospital in the morning and given a meal. Venous blood was collected for basal glycaemia, Insulin, C-peptide, and additionally for serum cortisol, catecholamines, GH and IGF-1, corresponding to time 0’, and every 6 h. Capillary blood glucose (CBG) was measured regularly, and in case of glycaemia < 60 mg/dL, frequency of venous sampling would increase to every 2 h. The test would be interrupted when Whipple triad was documented, if glycaemia was ≤45 mg/dL or when the fast had reached 72 h. At the end of the fast 1.0 mg of glucagon would be injected intravenously and glucose measured 10, 20, and 30 min later. The short fasting test (practiced since 2019) was performed as follows: the procedure was explained in full and consented by the patient. The fast would be initiated by the patient the evening before the evaluation, at home, with the last food intake at 07:30 pm, consisting of a meal with 70 grams of complex carbohydrates. All patients were previously instructed to restrain from physical activity, monitor symptoms and capillary glucose level and to be accompanied by a family member until admission to the hospital, in Day-Hospital regimen. On admission to the clinic at 08:00 a.m. at the following day (after a 12 h fast), CBG was measured with a point of care glucometer and a blood sample taken for glucose, insulin, C-peptide, cortisol, GH, IGF-1 and catecholamines. The patients were strictly monitored, and blood samples taken every 3 h for the next 12 h (up until 20:00 pm). In case of CBG < 60 mg/dL, finger-stick glucose sampling would be intensified to every 30 min to detect any significant descent; in case of CBG approaching 45 mg/dL or detection of neuroglycopenic symptoms, the test would be suspended. Otherwise, if the patient would be asymptomatic and with stable CBG, the fast would proceed with close finger-stick glucose monitoring every 30 min and venous sampling every 2 h. We proceeded with the fast in the above conditions due to the need to document glycaemia <55 mg/dL in order to reach threshold diagnostic values for insulinoma (ESCP Guidelines, 2009) [10], while minimizing the risk of inducing dangerous hypoglycaemia. Blood tests were determined through CLIA (*Architect*).

### Data analysis

Descriptive statistics were computed to all of the variables of interest. Associations between variables were obtained using Pearson’s Chi-Square test, with *p* < 0.05 considered as statistically significant. Statistical analyses were carried out using SPSS software (SPSS Inc., Version 29, Chicago, IL, USA).

## Results

The final sample consisted of 37 subjects, with mean age of 44.5 ± 12.6 years (17–74), 30 (81.0%) female e 7 (19.0%) male. Short and standard test were performed in 15 and 22 subjects, respectively. Diagnostic values for insulinoma were observed in 12/37 (32.4%; 11 female e 1 male), subsequently confirmed by imaging studies or histology in case of patients surgically treated. Among patients with insulinoma, 6 underwent the prolonged fasting test as first line, 5 underwent only the short test, while 1 patient underwent initially the short test with a negative result and subsequently the prolonged test with a positive result. Among patients with insulinoma, average age ± SD was of 48.4 ± 9.9 years while in the non-insulinoma group age was 42 ± 14.1 (*p* = 0.16). The majority of patients in both groups were female: 11/12 (91.7%) in the insulinoma group and 19/25 (76.0%) in the negative group (*p* = 0.38). No differences were observed among the two groups for BMI (26.2 ± 4.4 vs 26.1 ± 8.4 kg/m^2^; *p* = 0.33). Among the patients diagnosed with insulinoma, baseline mean values of glycaemia were 48.0 ± 11.47 mg/dL (range 29–64), with insulin 7.45 ± 3.17 µU/mL and C-peptide 2.04 ± 0.69 ng/mL. The remaining subjects negative for insulinoma had a significantly higher baseline glycaemia, with average 78.53 ± 14.04 mg/dL (range 58–119) (*p* < 0.001) along with lower insulin (mean 5.15 ± 3.6 µU/mL, *p* = 0.055) and significantly lower C-peptide (1.51 ± 0.66 ng/mL, *p* = 0.023). In patients with insulinoma, the mean duration of symptoms was 40 months. Both neuroglycopenic and adrenergic symptoms were reported in all 12 subjects (100%). Two patients reported weight gain and only 1 was obese grade I (BMI 30 kg/m2). Regarding concomitant pathological conditions in patients with insulinoma, 6/12 (50%) had thyroid disease. Of those, 5 were hypothyroid in replacement therapy with levothyroxine and one with selenium supplementation. One subject had a history of bariatric surgery. Other diagnosed pathologies in the insulinoma group included: uterine fibromatosis (3), osteoporosis (1), epilepsy (1), 1 patient had a history of papillary thyroid cancer and 1 patient had a previous diagnosis of a pancreatic non-functioning NET G2. No known cardiovascular, neurologic, psychiatric, gastrointestinal or other neoplastic conditions were recorded. In the group negative for insulinoma (25 patients), the duration of symptoms was 35.3 months as average. In contrast to the insulinoma group, neuroglycopenic symptoms were rarely reported, and were non-specified. Weight gain was reported in 2 patients, and 2 were obese.

In a total of 10/12 (83.33%) patients, the diagnosis of insulinoma was achieved within the 14^th^ hour of fasting, in 11/12 within 24 h (91.7%), while only one patient was diagnosed at more than 24 h (at 30^th^ hour). In the insulinoma group, minimum values of glycaemia registered during the fast for each subject (corresponding to the time of diagnosis) ranged from 28 to 50 mg/dL with concomitant insulinemia values ranging from 3.4 to 14.3 µU/mL and C-peptide from 0.60 to 3.3 ng/mL (Table 1).

**Table 1:** Characteristics of insulinoma patients, glycaemia at diagnosis with concomitant insulin and c-peptide (*ST: Short Fasting Test, PT: Prolonged Fasting Test)

Patient	Sex (m/f)	Age (years)	Test performed (ST/PT)*	Time to diagnosis (hours)	Glycaemia (mg/dL)	Insulinemia (mUI/mL)	C-Peptide (ng/mL)
1	f	54	ST	12	29	7.2	2.9
2	m	45	ST	13	44	6.4	1.1
3	f	35	ST	12	31	10.2	2.9
4	f	60	ST	12	50	12.7	2.1
5	f	37	ST	14	49	8.5	2.2
6	f	47	PT	4	28	8.0	3.3
7	f	55	PT	6	38	4.1	1.9
8	f	45	PT	6	42	4.3	1.2
9	f	63	PT	8	38	4.6	1.3
10	f	61	PT	9	44	10.4	2.4
11	f	37	PT	30	50	3.4	0.6
12	f	42	ST + PT	24	43	14.3	1.9

At admission to Day Hospital following the overnight home fast as part of the short fasting test, there was no record of important neuroglycopenic symptoms or any complications from hypoglycaemia, although two subjects presented asymptomatic critical hypoglycaemia, with CBG of 42 and 34 mg/dL; in these cases, the fast was immediately interrupted, venous blood collected and reversal of hypoglycaemia was verified rapidly after food intake.

The final diagnosis of patients with negative short and prolonged fasting tests (*n* = 25) were the following: normal glycaemic and insulinemic response to the fasting test was the final diagnosis in 6 patients (24%) with negative 72 h fast; cortisol/GH deficiency in 6 patients (24%), idiopathic reactive hypoglycaemia in 8/25 (32%), early unknown type 2 diabetes in 1 patient (4%), post gastric bypass hypoglycaemia with associated dumping syndrome in 2 patients (8%), hypoglycaemia through autonomic dysfunction in one patient with Arnold-Chiari syndrome (4%) and epilepsy in 1 patient (4%). Among the six patients diagnosed with deficiency of counterregulatory hormones, a preliminary 1-mcg ACTH stimulation test was normal in three, of whom one subsequently underwent the short fasting test and two underwent the standard fasting test. All were re-evaluated along the follow-up revealing a cortisol deficiency in two at the 1 mcg-ACTH test, while a diagnosis of GH deficiency in the remaining one at the GHRH test. Three other patients who presented baseline cortisol and ACTH within range and no signs of cortisol deficiency underwent the short fasting test first. They were diagnosed as cortisol deficient at the 1-mcg ACTH test performed in the subsequent follow-up.

## Discussion

Hypoglycaemic disorders may be classified according to their pathogenesis, which can be insulin mediated or non-insulin mediated. In apparently healthy individuals, hypoglycaemia is more likely due to endogenous hyperinsulinism which could be caused most frequently by an insulinoma and typically occurs in the fasting period, but could also derive from non-insulinoma pancreatogenous hypoglycaemia syndrome (NIPHS), insulin autoimmune hypoglycaemia, post bariatric surgery (dumping syndrome), accidental or fictitious hypoglycaemia (intake of beta cell secretagogues such as sulfonylurea). Idiopathic reactive hypoglycaemia (IRH), which occurs in the post-prandial state, has been associated with delayed and excessive second phase insulin response, an increased insulin sensitivity, or imbalance in incretin secretion, although underlying mechanisms are still not completely clear [10, 20, 21]. Conditions causing non-insulin mediated hypoglycaemia include states of critical illness, cortisol or GH deficiency, drugs, and certain malignant disorders (through production of IGF-I, IGF-II or pro-IGF-II) [10, 22]. In patients presenting with a history of hypoglycaemia and documented Whipple triad, diagnosis can usually be established by a laboratory assessment at the time of the spontaneous hypoglycaemic episode. When it is not observed by clinicians, a prolonged supervised fasting test, which can last up to 72 h, has been established as the gold-standard method for the assessment of the role of insulin secretion pattern in hypoglycaemia occurring in the fasting period. Through several series of cases of insulinoma, the Mayo clinic experience [14] concluded that the 72 h test was necessary to exclude false negative results in the few patients diagnosed on the 3^rd^ day of the fast. Nevertheless, a shorter modality of the test has been proposed by different authors, due to its practicality and potential first-line screening test: Hirshberg and colleagues [16] demonstrated that close to 100% of patients with endogenous hyperinsulinemia were diagnosed in less than 48 h. In their study the fast was terminated when plasma glucose was < 45 mg/dL in 43% patients within 12 h, in 67% within 24 h and in 95% within 48 h. Frajans e Vinik (1989) [17] evaluated 82 patients with endogenous hyperinsulinism caused by beta cell disorders: in blood samples obtained following a 12-h overnight fast in several different days, 76% patients showed glycaemia ≤ 50 mg/dL and in 86% ≤60 mg/dl. Vezzosi and colleagues [18] applied the same protocol: in their study, 88% of the subjects showed glycaemia < 60 mg/dL, concluding that it could be used as triage do exclude subjects without endogenous hyperinsulinemia. Moreover, Felicio and colleagues [19] achieved 100% of sensitivity in the diagnosis of hyperinsulinemic hypoglycaemia in blood samples of an overnight fast in 3 consecutive days. In our series, instead, 83.3% of insulinoma patients were diagnosed within 14 h and 91.7% within 24 h. In comparison to the above-mentioned protocols alternative to the gold standard 72 h fast, our protocol has the advantage of being potentially safer since it does not require leaving home after an overnight fast in several days to perform laboratory testing, instead it proceeds the overnight fast on one occasion in a monitored setting (Day Hospital) where hypoglycaemia can be promptly identified and reversed. Our results showed that the overnight fasting glycaemia was significantly lower and corresponding serum C-peptide significantly higher in the insulinoma group, (*p* < 0.001 and *p* = 0.023) as expected, which is in accordance with the current literature [1, 4]. The difference in baseline serum insulin, although being higher in the insulinoma group did not reach statistical significance (*p* = 0.055). Our preliminary results indicate that the short fasting test when portrayed up to 24 h could reach an excellent sensitivity for diagnosis of insulinoma (91.7%). False positive results deriving from other causes of endogenous hyperinsulinemia as insulin antibodies or NIPHS were not observed. It is also important to consider that during the short fasting test the biochemical evaluation starts at baseline after the overnight fast, 12 h from the last meal, therefore it is not possible to access glycaemia and insulinemia in the preceding time period. Given the likelihood that some patients show diagnostic values earlier, the real time to diagnosis is expected to be even shorter. Furthermore, there was a higher prevalence of the female sex in the insulinoma group (91.7%) in comparison to the non-insulinoma group (76.0%) without reaching statistical significance (*p* = 0.38). In current literature, some studies also reported a slightly higher prevalence of the female sex [2, 4]. Age and BMI were similar between insulinoma and non-insulinoma groups, which was also in accordance with literature [1]. Importantly, in consideration of the potential risk of dangerous levels of hypoglycaemia in insulinoma patients until arrival to hospital, we observed that the overnight fast was generally well tolerated, and there was no record of complications, which supports the safety of the procedure. In any case, we strongly recommended that the patient monitors symptoms closely and that should be always accompanied by a care giver until admission to the hospital. We do not recommend proceeding with the fast in case of neuroglycopenic symptoms at awakening. Finally, it is necessary to consider the cost-efficacy of both types of tests. The prolonged fasting test requires the availability of a bed for an elective hospitalization for up to 72 h, which in the actual post-pandemic setting, particularly in public hospitals across Europe, is not easily obtainable. The short fasting test reveals to be a more cost-effective alternative, as it is performed in Day Hospital/outpatient setting. The main limitations of this study are the retrospective design and the small sample size.

## Conclusions

In our series, more than 90% of patients with insulinoma were diagnosed within 24 h from the beginning of the fast. The short fasting test could be a valid, easily-manageable and reliable first-line work-up in diagnosing insulinoma. A prospective study is mandatory to confirm and further investigate this preliminary observation.
